# Voluntary Medical Male Circumcision for HIV Prevention in Swaziland: Modeling the Impact of Age Targeting

**DOI:** 10.1371/journal.pone.0156776

**Published:** 2016-07-13

**Authors:** Katharine Kripke, Velephi Okello, Vusi Maziya, Wendy Benzerga, Munamato Mirira, Elizabeth Gold, Melissa Schnure, Sema Sgaier, Delivette Castor, Jason Reed, Emmanuel Njeuhmeli

**Affiliations:** 1 Health Policy Project, Avenir Health, Washington, DC, United States of America; 2 Ministry of Health, Mbabane, Swaziland; 3 U. S. Agency for International Development (USAID), Mbabane, Swaziland; 4 Johns Hopkins Center for Communication Programs, Baltimore, MD, United States of America; 5 Health Policy Project, the Palladium Group, Washington, DC, United States of America; 6 Bill & Melinda Gates Foundation, Seattle, WA, United States of America; 7 Department of Global Health, University of Washington, Seattle, WA, United States of America; 8 Jhpiego, Baltimore, MD, United States of America; 9 USAID, Washington, DC, United States of America; University of Pittsburgh, UNITED STATES

## Abstract

**Background:**

Voluntary medical male circumcision (VMMC) for HIV prevention has been a priority for Swaziland since 2009. Initially focusing on men ages 15–49, the Ministry of Health reduced the minimum age for VMMC from 15 to 10 years in 2012, given the existing demand among 10- to 15-year-olds. To understand the implications of focusing VMMC service delivery on specific age groups, the MOH undertook a modeling exercise to inform policy and implementation in 2013–2014.

**Methods and Findings:**

The impact and cost of circumcising specific age groups were assessed using the Decision Makers’ Program Planning Tool, Version 2.0 (DMPPT 2.0), a simple compartmental model. We used age-specific HIV incidence from the Swaziland HIV Incidence Measurement Survey (SHIMS). Population, mortality, births, and HIV prevalence were imported from a national Spectrum/Goals model recently updated in consultation with country stakeholders. Baseline male circumcision prevalence was derived from the most recent Swaziland Demographic and Health Survey. The lowest numbers of VMMCs per HIV infection averted are achieved when males ages 15–19, 20–24, 25–29, and 30–34 are circumcised, although the uncertainty bounds for the estimates overlap. Circumcising males ages 25–29 and 20–24 provides the most immediate reduction in HIV incidence. Circumcising males ages 15–19, 20–24, and 25–29 provides the greatest magnitude incidence reduction within 15 years. The lowest cost per HIV infection averted is achieved by circumcising males ages 15–34: $870 U.S. dollars (USD).

**Conclusions:**

The potential impact, cost, and cost-effectiveness of VMMC scale-up in Swaziland are not uniform. They vary by the age group of males circumcised. Based on the results of this modeling exercise, the Ministry of Health’s Swaziland Male Circumcision Strategic and Operational Plan 2014–2018 adopted an implementation strategy that calls for circumcision to be scaled up to 50% coverage for neonates, 80% among males ages 10–29, and 55% among males ages 30–34.

## Introduction

Voluntary medical male circumcision (VMMC) has been shown to reduce female-to-male transmission of HIV by 60% [1–3] and is a recommended HIV prevention strategy in countries with high HIV prevalence and low levels of male circumcision [4]. Swaziland is one of 14 countries in eastern and southern Africa scaling up VMMC for HIV prevention following the recommendations of the World Health Organization (WHO) and the Joint United Nations Programme on HIV/AIDS (UNAIDS) [5]. With an HIV prevalence of 31% and HIV incidence of 2.4% among adults ages 18–49 years, Swaziland has the highest HIV prevalence and incidence in the world [6]. At the same time, with a male circumcision rate of 8.2% in its most recent Demographic and Health Survey, Swaziland has one of the lowest levels of male circumcision among the 14 VMMC priority countries [7].

In 2008, Swaziland developed the Policy on Safe Male Circumcision for HIV Prevention and the Strategy and Implementation Plan for Scaling up Safe Male Circumcision for HIV Prevention 2009–2013 [8,9]. According to the strategy, a key objective was to provide VMMC services to 144,688 HIV-negative males during the period 2009–2013 (111,688 males ages 15–24, and 33,000 neonates). Males ages 15–24 were selected as the primary population, because they were believed to be at greatest risk of infection [7,8]. In 2011, the Accelerated Saturation Initiative (ASI), also known as *Soka Uncobe* (meaning circumcise and conquer in SiSwati), was launched with the goal of circumcising 80% of males ages 15–49 within one year. While the ambitious ASI target was not met, important lessons were learned, especially about demand creation and the need to have strong community engagement and government leadership and coordination. Routine program data revealed important variation in demand for VMMC by age. Even though the program targeted 15- to 49-year-old males, there was significant demand for circumcisions among 10- to14-year-olds. Whereas clients ages 10–24 years comprised 75% of the client population between the start of the program in 2009 and the end of 2014, clients ages 25 years and above made up a lower proportion of clients than would be expected, based on the age distribution of uncircumcised males in the overall population. Fewer than 5% of clients were above the age of 35. In response to the demand for circumcision among 10- to 15-year-olds, the Ministry of Health decided to reduce the minimum age for VMMC from 15 to 10 years in 2012.

The Swaziland Ministry of Health (MOH) was interested in understanding the implications of focusing VMMC service delivery on specific age groups and aligning their VMMC targets with their implementation experience highlighting the heterogeneous demand among different age groups. In 2013/2014, the Decision Makers’ Program Planning Tool, Version 2.0 (DMPPT 2.0) was applied in Swaziland to assess the impact and cost of circumcising specific client age groups, and to inform the development of the country’s Male Circumcision Strategic and Operational Plan for HIV Prevention, 2014–2018 [10]. This paper describes the modeling done and the key policy decisions made based on the modeling results.

## Methods

### Overview of DMPPT 2.0

The DMPPT 2.0 model is described in detail elsewhere [11]. Briefly, DMPPT 2.0 is a simple, compartmental model implemented in Microsoft Excel 2010 that is designed to analyze the effects of age at circumcision on program impact and cost-effectiveness. The DMPPT 2.0 model tracks the number of circumcised males in newborns and in each five-year age group over time, taking into account age progression and mortality. The model calculates discounted VMMC program costs and HIV infections averted in the population in each year in a user-specified VMMC scale-up strategy, compared with a baseline scenario in which the male circumcision (MC) prevalence remains the same. The baseline scenario assumes that traditional or other circumcisions that produced the baseline MC prevalence continue at the same rate as before the VMMC program was initiated.

### Data used

The DMPPT 2.0 model is populated with population, mortality, and HIV incidence and prevalence projections from an external source. All model inputs are available in S1 Appendix and described here. For Swaziland, we used the age-specific HIV incidence from the Swaziland HIV Incidence Measurement Survey (SHIMS) for each year included in the analysis [6]. Because the SHIMS study did not include participants under the age of 18, while the DMPPT 2.0 model requires incidence by five-year age groups, the incidence among 10- to 14-year-olds was assumed to be zero and the incidence for the 15- to 19-year-olds was assumed to be 70% of the incidence among the 18- to 19-year age group from the SHIMS study. Incidence among men above age 49 was derived from the Goals model, because SHIMS incidence was also not available for those age groups. The population by age and year, mortality by age and year, annual number of male births, and HIV prevalence by age and year were imported from a national Spectrum/Goals model recently updated in consultation with country stakeholders [11,12].

Numbers of VMMCs conducted in the country, disaggregated by year and age group, were provided by the Ministry of Health on July 29, 2015. The MC prevalence by age group in the model base year (2014) was derived from the most recent Demographic and Health Survey [7], adjusted to reflect increases in MC coverage caused by the VMMC program since 2009. The unit cost of VMMC used in the analysis was $109 U.S. dollars (USD), based on an analysis conducted by Population Services International (PSI)/Swaziland, a VMMC implementer in the country (all subsequent references to currency are USD). VMMC unit cost was assumed to be constant across age groups. The annual per-person cost of antiretroviral therapy (ART) was $536, based on an ART costing study conducted in Swaziland in 2012 [13].

### Scenarios Analyzed

To examine the effect of client age on the impact of scaling up VMMC, we created a series of scenarios. Each scenario had a target of 80% MC coverage for a single age group or combination of age groups, leaving the target for the other age groups at the same level as the baseline. We created one scenario for each individual five-year age group, in addition to several scenarios with 80% targets for combined age groups, such as 10–34 or 15–29. Each scenario scaled up MC coverage between 2014 and 2018, by applying a linear interpolation to the baseline MC prevalence for each age group in 2013 and the target coverage in 2018. After 2018, the coverage for each age group was maintained at the target level. For each scenario we compiled the decrease in HIV incidence in the scale-up scenario compared with the baseline scenario in each year of the model, and the total number of circumcisions required during the scale-up phase (2014 to 2018).

The following model outputs for each scenario were measured over the 15-year period between 2014 and 2028, inclusive: the total number of HIV infections averted in the population (including secondary infections averted among females, because, although the primary impact of VMMC is on the number of new HIV infections in men, HIV infection in women is also affected through a reduced probability that a woman will come into contact with an HIV-infected man); the number of VMMCs per HIV infection averted; and the total cost of the VMMC program. Costs, numbers of circumcisions, and infections averted were all discounted at a rate of 3% per year. Uncertainty around the age distribution of HIV incidence was calculated as described in [11].

We assessed VMMC age-targeting strategies according to the four following metrics: the number of VMMCs per HIV infection averted over a period of 15 years or the efficiency of the strategies; the reduction in HIV incidence in the near term (five years) or the immediacy of impact; the reduction in HIV incidence in the medium term (15 years) or the magnitude of impact; and the cost-effectiveness of the strategies, measured as the cost per HIV infection averted over 15 years [11].

## Results

Kripke et al. [11] show that when assessing all five-year age groups starting with age 10, the lowest numbers of VMMCs per HIV infection averted between 2014 and 2028 were produced by circumcising males ages 15–19 (mean: 12, uncertainty: +/- 4), 20–24 (mean: 8, uncertainty: +/- 2.3), 25–29 (mean: 8, uncertainty: +/- 2.8), and 30–34 (mean: 9, uncertainty: +/- 4.3). The uncertainty bounds for these estimates overlap, due to the wide confidence intervals around the HIV incidence estimates from the SHIMS study (S1 Fig).

**Fig 1:**
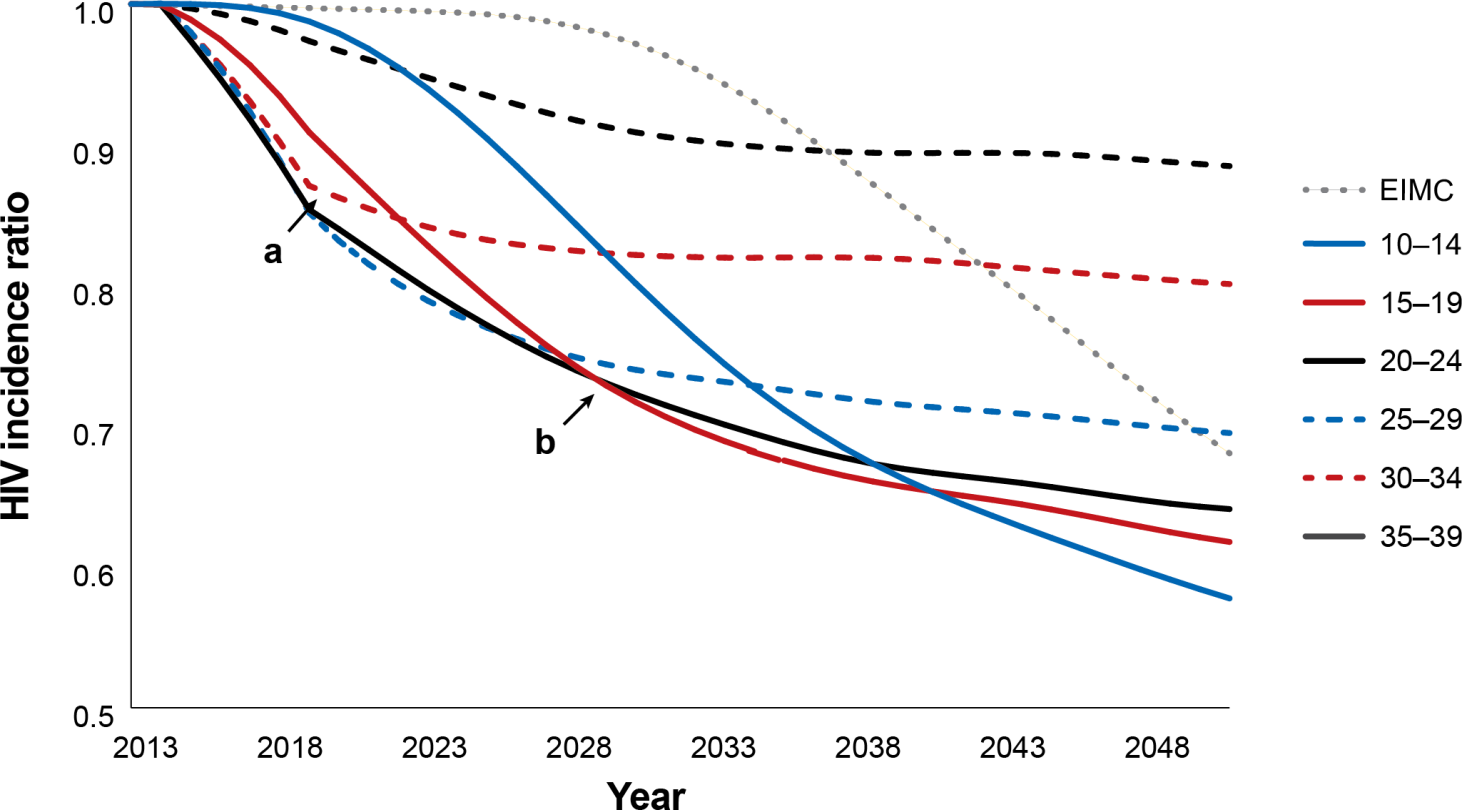
Reduction in HIV incidence with provision of VMMC to males, by age group, 2014–2050. The HIV incidence ratio represents the incidence in the scale-up scenario divided by the incidence in a population where circumcision is not scaled up over baseline levels. HIV incidence is in the entire population—males and females. Each line represents the HIV incidence ratio under a scenario in which only the indicated five-year age group is circumcised. Marker ***a*** indicates a point five years from the base year (2014). Marker ***b*** indicates a point 15 years from the base year.

Fig 1 depicts the relative impact of circumcising different age groups on HIV incidence over time. Each line corresponds to a hypothetical scenario in which circumcision coverage was scaled up within one five-year age group alone and the number of HIV infections averted were projected over the entire population. When considering only the five-year period 2014–2018 (immediacy of impact), the most rapid reductions in incidence can be seen in the strategies focused on the 20–24 and 25–29 year age groups. The 30–34 year age group initially presents a similar decline, but it levels off earlier. In contrast, when considering a longer time frame, the reduction in HIV incidence flattens out at a level related to the fraction of lifetime protection conferred by circumcising at a given age. The greatest reductions in incidence after 15 years (magnitude of impact) can be seen in the 15–19, 20–24, and 25–29 year age groups in Swaziland. When considering an even longer perspective (2014–2048), circumcising males in the youngest age group (ages 10–14) confers the greatest long-term benefits, as these males are protected from HIV for their entire lifetime of sexual exposure to HIV, and these benefits accrue over time to the males themselves and to their sexual partners. Conversely, males circumcised in the higher age groups have less time to benefit from the protection of circumcision. The impact of early infant male circumcision (EIMC, circumcising infants before the age of 60 days) is delayed by 15 to 18 years, the time that it takes for the boys to reach sexual maturity.

**Fig 2:**
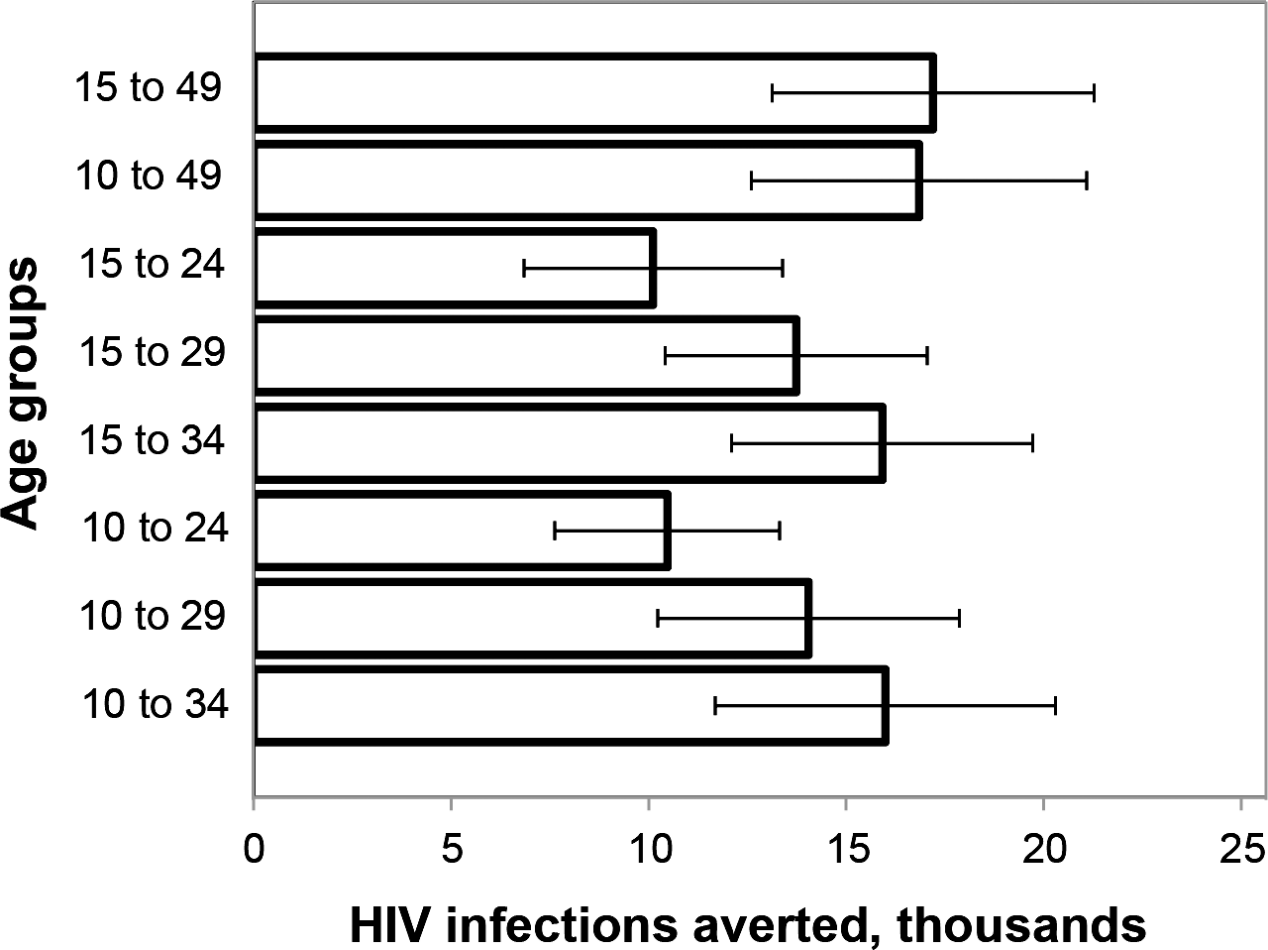
HIV infections averted in scenarios scaling up VMMC among different client age groups. The period for measuring HIV infections averted was 15 years, 2014–2028, inclusive. Error bars represent uncertainty bounds.

The impact of scaling up VMMC among broader age groups that might represent actual implementation strategies (contiguous age groups versus discrete five-year age groups) is shown in Fig 2. A scenario scaling up VMMC to 80% coverage among males ages 15–49 is used as a reference, since this represents Swaziland’s VMMC scale-up target prior to initiating this analysis. In general, the broader the age group included (i.e., the more males are circumcised), the greater the impact will be. If a narrower age group is needed due to practical constraints, scaling up VMMC among males ages 10–34 results in 93% of the HIV infections averted compared with scaling up among males ages 10–49. Although again, we note the overlap in the uncertainty bounds.

**Fig 3:**
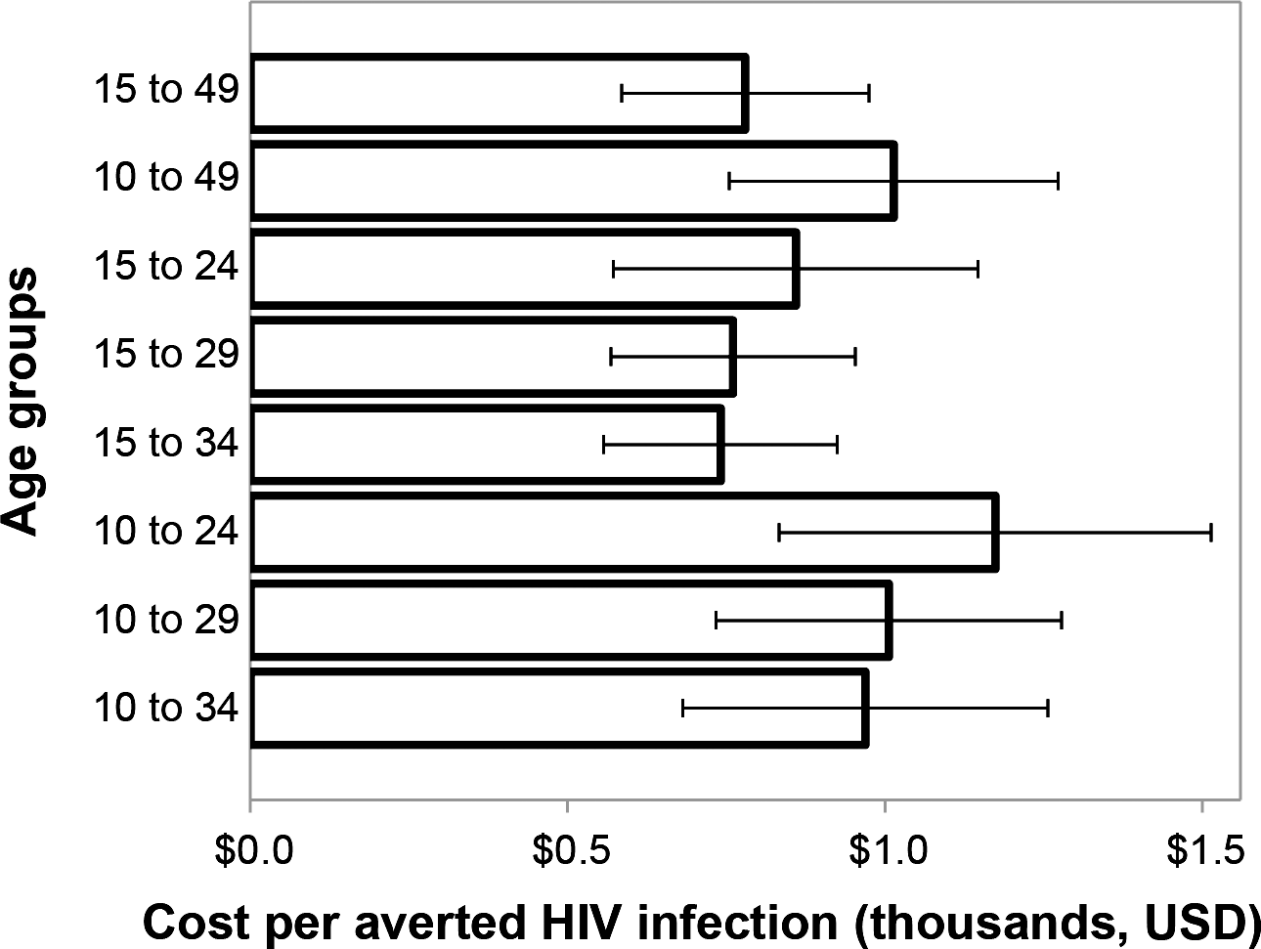
Cost per HIV infection averted in scenarios scaling up VMMC among different client age groups. The period for measuring HIV infections averted was 15 years, 2014–2028, inclusive. Error bars represent uncertainty bounds.

Fig 3 shows that the lowest cost per HIV infection averted over a 15-year time horizon is achieved by circumcising males ages 15–34 ($870) and 15–29 ($890), using the same VMMC unit cost across all age groups. As with Fig 2, the uncertainty bars largely overlap. The scenarios with age 10 as the lower age bound cost more per HIV infection averted by 2028 than the corresponding scenarios with 15 as the lower age bound.

We also analyzed the six scenarios outlined in Table 1 for their projected impact, cost, and cost-effectiveness (Table 2). Scenarios D, E, and F were developed to be consistent with the goals of the Extended National Multisectoral HIV and AIDS Framework 2014–2018 of scaling up to 70% the overall MC coverage among males ages 10–49 by 2018 and circumcising 50% of infants before the age of 60 days (EIMC) [14]. Scenario C, which set a target of scaling up EIMC to 50% coverage and scaling up to 80% circumcision coverage among males ages 10–34, was projected to have the highest number and percentage of HIV infections averted and the lowest cost per HIV infection averted, but also the highest total cost. Scenario A, which set a target of scaling up EIMC to 50% coverage and scaling up to 80% circumcision coverage among males ages 10–24, was projected to have the lowest total cost, but also the lowest number of HIV infections averted and the highest cost per HIV infection averted. It also reached only 59% circumcision coverage among males ages 10–49. Scenarios D, E, and F were suggested by country stakeholders based on what they expected would be more feasible to implement. Of these, Scenario E, which set a target of scaling up EIMC to 50% coverage and scaling up to 80% circumcision coverage among males ages 10–29 and 55% circumcision coverage among males ages 30–34, resulted in the greatest number of infections averted for the lowest cost.

**Table 1:** Swaziland Age-targeting Strategy Scenarios.

Scenario	Baseline	A (50% EIMC and 80% 10–24)	B (50% EIMC and 80% 10–29)	C (50% EIMC and 80% 10–34)	D	E	F
EIMC	0%	50%	50%	50%	50%	50%	50%
10–14	4%	80%	80%	80%	80%	80%	90%
15–19	4%	80%	80%	80%	80%	80%	90%
20–24	7%	80%	80%	80%	80%	80%	80%
25–29	8%	8%	80%	80%	70%	80%	70%
30–34	10%	10%	10%	80%	60%	55%	35%
35–39	20%	20%	20%	20%	50%	20%	20%
40–44	13%	13%	13%	13%	40%	13%	13%
45–49	12%	12%	12%	12%	30%	12%	12%
50–54	12%	12%	12%	12%	12%	12%	12%
55–59	12%	12%	12%	12%	12%	12%	12%

The first column shows the age groups. Percentages indicate the target male circumcision coverage in each age group. The baseline column shows the baseline circumcision prevalence in each age group before the VMMC program was started, based on the Swaziland Demographic and Health (DHS) Survey 2006–2007.

**Table 2:** Impact, Cost, and Cost-effectiveness of VMMC Scale-up Scenarios, 2014–2028.

Scenario	A	B	C	D	E	F
MC coverage among males ages 10–49	59%	68%	75%	70%	70%	70%
HIV infections averted (thousands)	20 (14, 24)	27 (19, 34)	29 (21, 38)	27 (21, 33)	27 (21, 33)	25 (19, 33)
Total cost (millions, USD)	$29	$33	$36	$34	$34	$35
% HIV infections averted	47%	64%	72%	65%	66%	63%
VMMC per HIV infection averted	16 (12, 21)	13 (10, 17)	13 (9, 17)	13 (10, 17)	13 (10, 16)	14 (10, 18)
Cost per HIV infection averted	$1,500 ($1,100, $1,900)	$1,300 ($900, $1,600)	$1,200 ($900, $1,600)	$1,300 ($1,000, $1,600)	$1,300 ($900, $1,600)	$1,400 ($1,000, $1,700)

Figures in the table are rounded to two significant figures. Numbers in parentheses represent uncertainty bounds.

## Discussion

The modeling analysis detailed in this paper illustrates that the potential impact, cost, and cost-effectiveness of VMMC scale-up in Swaziland is not uniform. It varies by the age group of males circumcised.

Key model findings for Swaziland are as follows. The lowest numbers of VMMCs per HIV infection averted (over 15 years, 2014–2028) are achieved when males ages 15–19, 20–24, 25–29, and 30–34 are circumcised. Circumcising males ages 20–24 and 25–29 provides the most immediate reduction in HIV incidence (by 2018). Circumcising males ages 15–19, 20–24, and 25–29 provides the greatest magnitude incidence reduction within 15 years (by 2028). Circumcising males ages 10–14 provides the greatest incidence reduction when considering the very long term. In general, the broader the age group included (the more males are circumcised), the greater the impact will be. The impact among males ages 10–49 is slightly smaller than that among males ages 15–49, because of the effects of discounting. The lowest cost per HIV infection averted (the greatest cost-effectiveness, 2014–2028) is achieved by circumcising males ages 15–34 ($870). While not examined in detail in this paper, it should be clear from Fig 1 that all of these findings are dependent on the period over which the impact is assessed.

Because of the wide confidence intervals in the HIV incidence estimates from the SHIMS study, upon which the uncertainty bounds for the model estimates depend, there is overlap in the uncertainty bounds between age groups for the estimates for the numbers of VMMCs per HIV infections averted, the numbers of HIV infections averted, and the cost per HIV infection averted. (The total cost is not affected, as it does not depend on incidence but only on population projections, which were not varied in the uncertainty analysis.) Therefore, these results are not distinguishable according to this analysis. However, for model exercises for other country applications, which had much narrower uncertainty bounds around the model estimates due to different methodology, there was much less overlap in the model estimates [15–19].

Despite the wide uncertainty bounds, policymakers in Swaziland chose to base their revised age targeting strategy on the model results. The Extended National Multisectoral HIV and AIDS Framework 2014–2018 (an extension of the 2009–2014 National Strategic Framework for HIV/AIDS) had previously identified male circumcision as a priority program for its “maximum scenario package,” calling for 70% coverage of men ages 10–49 by 2018. In order to balance impact, cost, and program feasibility—while also aligning with this goal of 70% coverage—the Ministry of Health chose to use the DMPPT 2 modeling results to directly inform the circumcision targets and priorities within the Swaziland Male Circumcision Strategic and Operational Plan for HIV Prevention for 2014–2018. The country also used the model to set age-specific annual targets at the inkhundla (district) level.

The Ministry of Health chose to adopt Scenario E, which calls for circumcision to be scaled up to 50% coverage for neonates, 80% among males ages 10–29, and 55% among males ages 30–34 (see Table 1), for its Strategic and Operational Plan. This scenario maintains the mandated 70% coverage among males ages 10–49, while balancing cost, cost-effectiveness, and impact (Table 2). This strategy was also chosen for the feasibility of its implementation in Swaziland, in terms of which age groups of men were willing to be circumcised. The plan uses the DMPPT 2.0 results to highlight both the impact and cost-effectiveness of the proposed program, noting that it will avert over 31,000 new HIV infections by 2028 (over 56,000 by 2035) and will result in a discounted cost savings of approximately $370 million by 2035. (These numbers differ from those reported in this manuscript because the numbers in the Plan are not discounted, while those in this manuscript are discounted.)

It should be noted that EIMC was included in the scenarios analyzed, because circumcising 50% of infants is part of the eNSF target. EIMC adds to the costs of the program but does not contribute to infections averted over the 15-year period examined; it is included in the strategy as part of the plan for sustainability of the VMMC program and also because of its long-term impact.

The country chose age 10 as the lower bound for scenarios to consider, because of the large proportion of current VMMC clients ages 10–14 years. This was done even though over the time frame studied, inclusion of males ages 10–14 leads to only small increases in HIV infections averted and results in a higher cost per HIV infection averted. While an analysis focused on short-term impact might lead one to conclude that circumcising males ages 10–14 should not be a program priority, turning away circumcision clients is viewed as problematic by implementers, given the limited demand for VMMC in Swaziland to date among older age groups and the fact that circumcising 10- to 14-year-olds contributes to medium-term (15-year) and long-term (30-year) impact but not to immediate (5-year) impact.

## Limitations

Although the limitations of the DMPPT 2.0 model have been described elsewhere [11], the following additional limitations should be considered when interpreting these findings. First, the SHIMS incidence data were used for every year of the model; therefore, the model did not project any changes in HIV incidence over time. Depending on actual future HIV incidence trends, this will likely result in over- or underestimating the impact of circumcision on HIV incidence, with impact being greater with higher incidence trends, and vice versa. However, projected relative impact of circumcising the different age groups will remain the same as long as the age distribution of HIV incidence remains the same. Second, the VMMC unit cost was estimated based on PSI expenditure data, and was not a result of a facility-based costing study. We assumed that the VMMC unit cost was the same regardless of client, when in fact it is probable that the unit cost varies by client age, given that ease of reach varies by age.

While providing insight on the impact and cost of the age-targeting options for the VMMC program in Swaziland, this modeling does not provide information on *how* to scale up VMMC in Swaziland. It does not address the programmatic considerations critical to scale-up, including the demand creation necessary and its cost, or the human resource requirements—including the importance of task-shifting to providers in rural health centers, for example.

## Supporting Information

S1 Appendix Swaziland DMPPT 2.0 Model Inputs. See Methods section for data sources.
(XLSX)

S1 Fig Swaziland age-specific male HIV incidence from the Swaziland HIV Incidence Measurement Survey (SHIMS) [6, 20]. Vertical bars represent 95% confidence intervals.
(TIF)

## Abbreviations

USAID: United States Agency for International Development
VMMC: voluntary medical male circumcision
MOH: Ministry of Health
DMPPT: Decision Makers’ Program Planning Tool
SHIMS: Swaziland HIV Incidence Measurement Survey
USD: U.S. dollars
eNSF: Extended National Multisectoral HIV and AIDS Framework
WHO: World Health Organization
UNAIDS: Joint United Nations Programme on HIV/AIDS
ASI: Accelerated Saturation Initiative
MC: male circumcision
PSI: Population Services International
ART: antiretroviral therapy
EIMC: early infant male circumcision
PEPFAR: President’s Emergency Plan for AIDS Relief
PPD ARO: Partners in Population and Development, Africa Regional Office
PRB: Population Reference Bureau
WRA: White Ribbon Alliance
DHS: Demographic and Health Survey
