# Correction: Voluntary Medical Male Circumcision for HIV Prevention in Swaziland: Modeling the Impact of Age Targeting

**DOI:** 10.1371/journal.pone.0169697

**Published:** 2017-01-03

**Authors:** Katharine Kripke, Velephi Okello, Vusi Maziya, Wendy Benzerga, Munamato Mirira, Elizabeth Gold, Melissa Schnure, Sema Sgaier, Delivette Castor, Jason Reed, Emmanuel Njeuhmeli

There is an error in the Funding statement. The number for the Cooperative Agreement Project SOAR (Supporting Operational AIDS Research) is incorrect. The correct number is OAA-A-14-00060.
