# Supplementary figures and images for: Voluntary Medical Male Circumcision for HIV Prevention in Swaziland: Modeling the Impact of Age Targeting
Source: PLoS One. 2016 Jul 13;11(7):e0156776. doi: 10.1371/journal.pone.0156776 (PMC4943626; doi:10.1371/journal.pone.0156776)

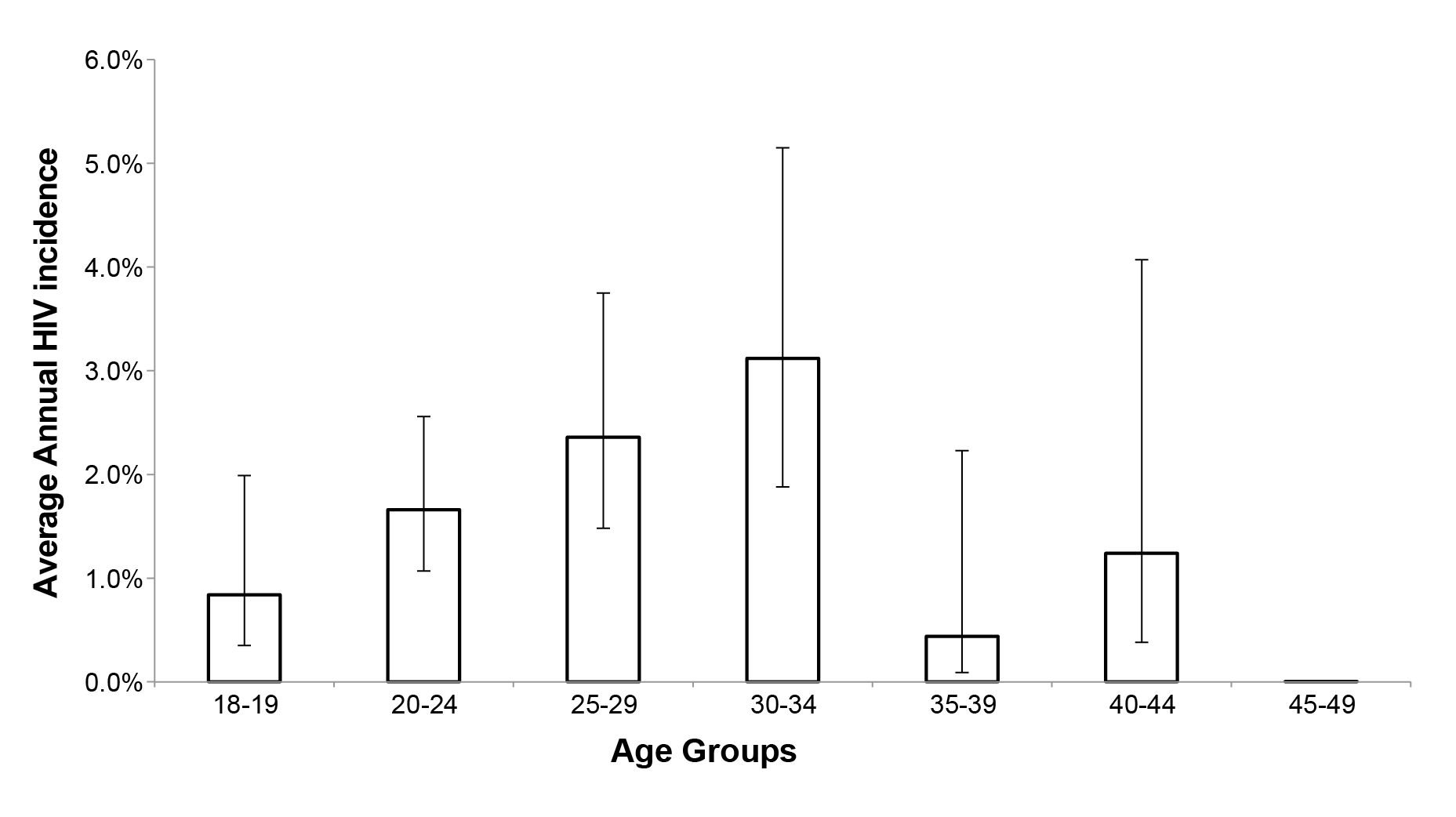

Supplement: S1 Fig — Vertical bars represent 95% confidence intervals. (TIF) [file pone.0156776.s002.tif]
